# Self-assembled monolayers of alendronate on Ti6Al4V alloy surfaces enhance osteogenesis in mesenchymal stem cells

**DOI:** 10.1038/srep30548

**Published:** 2016-07-29

**Authors:** Luis Rojo, Borzo Gharibi, Robert McLister, Brian J. Meenan, Sanjukta Deb

**Affiliations:** 1 Division of Tissue Engineering & Biophotonics, King’s College London Dental Institute. Guy’s Hospital, London Bridge, London, United Kingdom; 2 Nanotechnology and Integrated BioEngineering Centre (NIBEC), School of Engineering, University of Ulster, Northern, Ireland.

## Abstract

Phosphonates have emerged as an alternative for functionalization of titanium surfaces by the formation of homogeneous self-assembled monolayers (SAMs) via Ti-O-P linkages. This study presents results from an investigation of the modification of Ti6Al4V alloy by chemisorption of osseoinductive alendronate using a simple, effective and clean methodology. The modified surfaces showed a tailored topography and surface chemistry as determined by SEM microscopy and RAMAN spectroscopy. X-ray photoelectron spectroscopy revealed that an effective mode of bonding is created between the metal oxide surface and the phosphate residue of alendronate, leading to formation of homogenous drug distribution along the surface. *In-vitro* studies showed that alendronate SAMs induce differentiation of hMSC to a bone cell phenotype and promote bone formation on modified surfaces. Here we show that this novel method for the preparation of functional coatings on titanium-based medical devices provides osseoinductive bioactive molecules to promote enhanced integration at the site of implantation.

Titanium and medical grade Ti6Al4V alloy are the most prevalent materials used to fabricate dental and orthopaedic implants, primarily due to their suitable mechanical properties and intrinsic ability to support osseointegration^1^. The effectiveness of this process is governed by the interactions that occur at the outermost surface layer over a few atomic distances with the host extracellular matrix and local cell population. Thus, targeted biomimetic surface modification of implants can help to improve their integration with surrounding tissue and is accomplished by a number of established approaches, including the formation of self-assembled monolayers (SAMs)^2^,^3^. The key objective here is to direct the interaction between the host tissue and the implant towards a condition that supports a complete and successful osseointegration process^4^. An example of such a treatment consists of treatment with alkyl trichlorosilane derivatives that react with the titanate groups to form siloxane networks that can be further functionalised with different signalling molecules or bioactive peptide moieties to enhance their bioactivity^3^,^5^. However, it has been demonstrated that this methodology has significant problems in that it is difficult to control; susceptible to unwanted hydrolysis and can lead to the formation of non-uniform layered interfaces^6^. Organophosphorus compounds such as phosphonates have emerged as an alternative means of functionalization of titanium surfaces with homogeneous self-assembled monolayers (SAMs). When presented to the surface as organic dispersions they can bind via Ti-O-P bonds that are superior to that achieved by direct silanisation methods^7^,^8^.

The type of organic solvent based condensation reaction that occurs between phosphonates and metal surfaces has been studied extensively for applications such as anticorrosive and high lubricous coatings^9^. This approach utilises the conventional deposition of phosphonate film followed by the monolayer condensation reaction promoted by heat treatment. This methodology limits the application, especially for medical devices and implants, where the presence of even minor organic solvent residues cannot be tolerated. However, only few studies have investigated the capability of water soluble phosphonates to form self-assembling monolayers^10^,^11^ in a manner that might be used for biomedical coating applications.

Alendronate is a well-known bisphosphonate commonly used in osteoporosis therapy and treatment of other bone diseases^12^. Importantly, it has recently been incorporated into heparin modified titanium implants for local delivery^13^. However, to date it has not been directly immobilized onto titanium implants to form stable chemisorption monolayers that can functionalise the surfaces. Hence, this paper presents results from a study of the osseoinductive potential of alendronate SAMs on Ti6Al4V alloy substrates. This approach has not been explored earlier and suggests that the presence of alendronate and related SAMs overcome some of the limitations of current implantology associated to lack of osseointegration and systemic administration of additional bone enhancing drug.

## Results and Discussion

The quest to enhance the biological response of titanium and its alloys via alteration of the surface finish either through the introduction of coatings such as calcium phosphate or modifications such as electro-polishing, alone or in combination has continued over the past three decades^1^,^14^. More recently, the role of both the chemistry and topography of titanium implant surfaces in their osseointegration capability is being explored^15^. In this study, we report how a self-assembly monolayer of the bisphosphonate compound alendronate deposited onto Ti6Al4V alloy substrates with different surface topographies effect their bioactivity. The surface roughness of electro-polished (P-Unt) and Bioglass^®^ grit-blasted (GB-Unt) Ti6Al4V alloy substrates were obtained using white light profilometer measurements, with the values provided in Table 1. As expected, the data indicated that the roughness value increased slightly after the electro-polishing whilst the grit-blasting treatment with Bioglass^®^ significantly increased (p > 0.5) the average surface roughness (Ra) of the samples with Ra values > 1 μm. The contact angle values did not differ significantly between P-Unt and GB-Unt surfaces whilst the electro-polished alendronate SAM (P-ALE) coated samples showed a decrease as a consequence of the presence of ionisable hydroxyl and amino groups, which contribute to the polar component of surface free energy, thereby increasing the hydrophilicity (Table 1).

**Figure 1:**
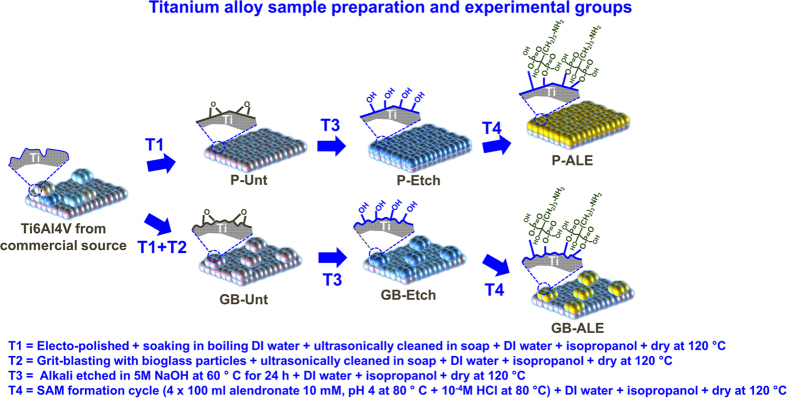
Schematic representation of the fabrication process for alendronate SAMs onto TiAl6V4 alloy indicating sample treatments and resulting surfaces electro-polished (P-Unt), electro-polished alkali etched (P-Etch), electro-polished alendronate SAM (P-ALE), grit-blasted (GB-Unt), grit-blasted alkali etched (GB-Etch) and grit-blasted alkali etched alendronate SAM (GB-ALE).

**Figure 2:**
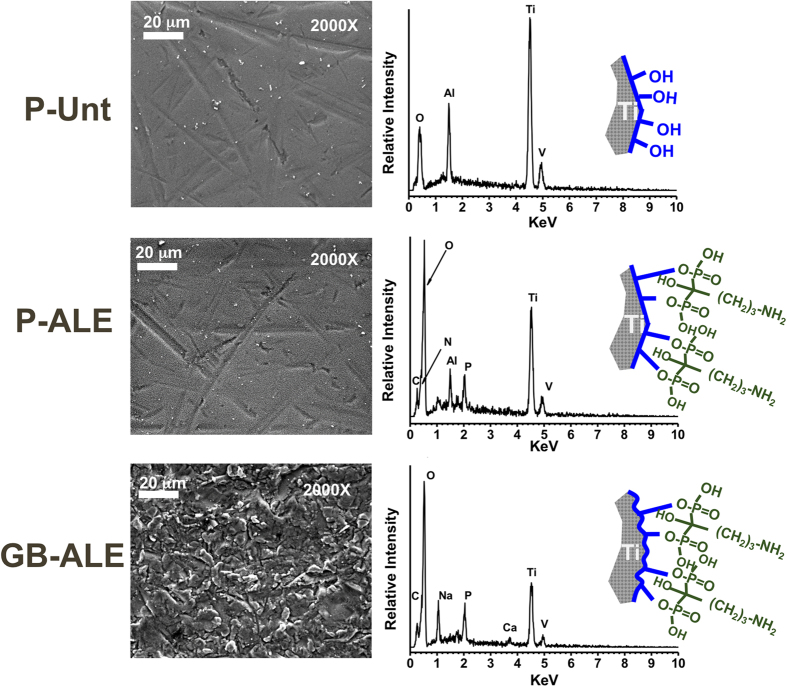
SEM and EDX analysis spectra for P-Etch, P-ALE and GB-ALE surfaces.

The condensation reactions that can occur between phosphonates and metal surfaces have been extensively studied earlier^8^,^10^ for different applications such as the provision of anticorrosive and high lubricous coatings. Conventional methodologies utilize a two-step procedure in which a weakly adsorbed Langmuir-Blodgett phosphonate film is formed in the first stage followed by the monolayer condensation reaction promoted by heat treatment. This procedure complicates the scaling up of the procedure and limits its application, especially in applications such as coatings for medical devices and implants, where the presence of even minor organic solvent residues in the ad-layers can result in adverse events. Hence, an alternative procedure is presented here in which the condensation reaction takes place after deposition of an aqueous solution of alendronate, which is subsequently heated to 80 °C on a hot plate in order to eliminate the solvent and condensed water formed during the phospho-esterification reaction. A scheme outlining the mechanism of this reaction is illustrated in Fig. 1. This thermal treatment induced mechanism has been demonstrated to produce an ordered film of alkyl phosphates that is strongly bound to the substrate through monodentate, bidentate, tridentate interactions or a mixture of these configurations^11^,^16^ but with relatively moderate conditions to ensure the thermal stability of alendronate^17^. Deposition of alendronate and resistant to hydrolysis were confirm gravimetrically giving a mean weight gain of 0.22 ± 0.02 mg (n = 8, mean ± mean standard error) for P-ALE substrates with no significant change after immersion in phosphate buffered saline (PBS) for 14 days (p < 0.05), thus evidencing the unappreciable release of the phosphate by hydrolysis. As such, it constitutes a non-hazardous and easily scalable procedure that ensures the creation of a homogenous alkyl phosphate composition and microstructure on the treated surface. High magnification scanning electron microscopy (SEM) images of Grit-Blasted etched (GB-Etch), P-ALE and Grit-Blasted Alendronate SAMs (GB-ALE) Ti6Al4V surfaces, are shown in Fig. 2, which indicate a distinct change in the substrate microstructure after the surface modification process. Chemical anchorage of the SAM coating to the metal alloy surface was confirmed by energy disperse X-ray (EDAX) analysis with new peaks arising at 0.28, 0.39 and 2.02 keV corresponding to the presence of C, N and P respectively. Additional peaks assigned to Na and Ca can be observed for GB-ALE substrate as consequence of remaining abrasive bioglass particles on the conditioned surfaces^18^.

**Figure 3:**
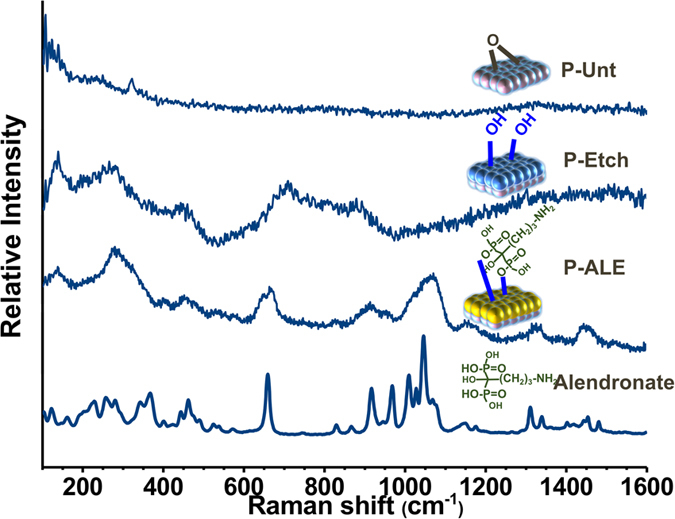
Raman spectra of P-Unt, P-Etch and P-Ale substrates. The scan for pristine alendronate drug from commercial source is included for reference.

To further confirm the bonding of alendronate SAM coating formed on the titanium alloy surface, RAMAN spectroscopy and X-ray photoelectron spectroscopy (XPS) analysis measurements were performed. Raman spectra for P-Unt surfaces before and after alkali etching, and after deposition of alendronate via the SAM treatment, are shown in Fig. 3. A spectrum of the alendronate solution used in the coating method is also included for comparison. These spectral data clearly indicate the change of surface composition that results after the different stages of surface treatment. The electro-polished etched (P-Etch) samples showed a Raman spectrum typical of the titanate Ti6Al4V with main peaks arising at 138 cm^−1^, 270 cm^−1^ and 449 cm^−1^, generally attributed to νTi-O vibrations of the anatase phase^19^,^20^, and a characteristic band at 720 cm^−1^ assigned to the νO = Ti-OH complex^21^. The presence of this hydroxyl layer alters the surface properties and results in change of the substrate wettability as indicated by the calculated surface free energy (SFE) increasing from 66.5 mN/m to 73.9 mN/m (Table 1), which then facilitates the subsequent chemisorption reaction between the modified Ti6Al4V and the alendronate solution. The formation of covalent bonding during the second step is evidenced by the further increase of the SFE to 79.5 mN/m and the disappearance of the 720 cm^−1^ band accompanied by the appearance of new peaks at 667 cm^−1^, 1069 cm^−1^, 1327 cm^−1^ and 1447 cm^−1^ that are characteristic of the νC-P, ν_as_O = P-O, νC-C and δCH_2_ vibration modes of alendronate^22^.

**Figure 4:**
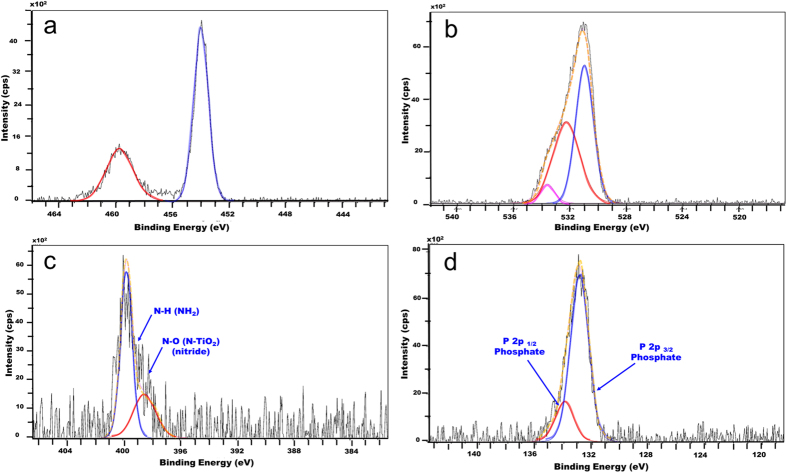
High-resolution XPS spectra for (**a**) Ti-2p, (**b**) O-1s, (**c**) N-1s, d: P-2p regions of GB-ALE substrates.

More information on the chemical state of the alendronate SAM layers on Ti6Al4V and the mode of bonding to the substrate was obtained through analysis of high resolution XPS spectra with regions of interest corresponding to the Ti-2p (~454 eV), O-1s (~530 eV), N-1s (~400 eV) and P-2p (~133 eV) signals obtained for P-Etch, P-ALE and GB-ALE substrates. The C1s (285 eV) signal was not considered for quantification purposes here due to the fact that that adventitious carbon on the titanium surfaces makes it difficult to accurately establish the alendronate contribution^23^. The P-Etch samples showed two characteristic O-1s signals (Supplementary Figure S1) at 531.1 eV (FWHM = 1.8 eV; 37.2%at) and 532.5 eV (FWHM = 2.3 eV; 10%at) that were assigned to the oxide (Ti-O_2_) and hydroxide (Ti-OH) bonding modes, respectively. The lack of any detectable N or P elements at their corresponding binding energies confirmed the absence of any organic compounds contaminants on this surface. On the contrary, *SAM* coatings on both the GB-ALE and P-ALE samples as shown in Fig. 4 and Supplementary Figure S2 respectively, showed three O-1s signals centred at 531.1 eV (FWHM = 1.6 eV; 27.4%at), 532.7 eV (FWHM = 1.7 eV; 12.5%at) and 534.0 eV (FWHM = 1.0 eV; 2.1%at) which relate to the presence of P-O-Ti, P = O and HO-C- bonds^10^,^11^,^24^ attributed to the presence of the bisphosphonate monolayer. In addition, the lack of a P-OH peak at 533 eV indicates a bidentate or tridentate bonding mode between the SAM and the substrate. The N-1s region reveals a main contribution at 399.9 eV (FWHM = 1.3 eV; 2.6%at) that corresponds to free C-NH_2_ from the alendronate residue and a small contribution at 398.5 (FWHM = 0.7 eV; 0.3%at) that can be assigned to a Ti-O-N oxidized nitride mode of binding^25^. In addition, characteristic asymmetric P-2p peaks, fitted as an unresolved P-2p_3/2_ and P-2p_1/2_ doublet, centred at 134.0 (FWHM = 1.5 eV; 2.1%at) and 133.1 (FWHM = 1.5 eV; 4.6%at) respectively, where observed confirming the presence of the bisphosphonate monolayer. Quantification data of the nitrogen (N-1s) and phosphorous (P-2p) content on these substrates are shown in Table 2. Calculation of an N/P ratio in the presence of the alendronate SAM coating of 0.5 indicates the absence of any hydrolysis or thermal degradation that might have occurred during the monolayer formation and therefore demonstrates the feasibility of this SAM formation methodology to anchor bioactive bisphosphonate from aqueous solution onto medical implants. The presence of the free amino pendant groups in the monolayer is of particular interest, as it offers the possibility to undertake further surface modification in a manner similar to that used for other coupling agents that are already in use for binding bioactive molecules to titanium surfaces such as Shift-base reaction or carbodiimide chemistry^18^.

**Figure 5:**
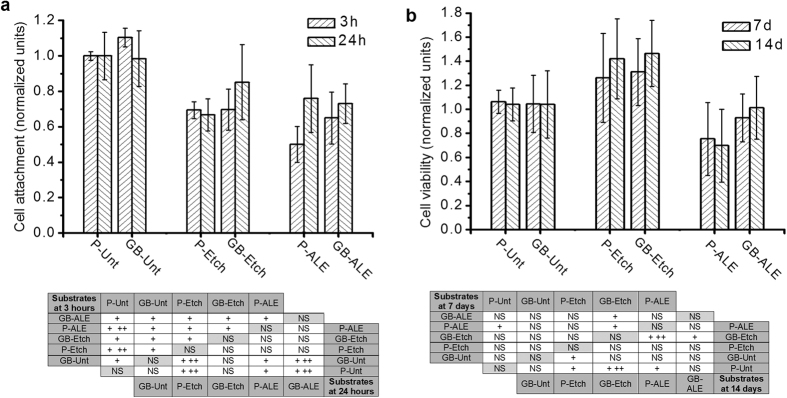
(**a**) Number of hMSCs attached on treated substrates, determined by MTS assay after 3 h and 24 h in culture and statistical analysis of significant differences between groups. (**b**) Viability of hMSCs on treated substrates, determined by MTS assay, after 7 days and 14 days in culture and statistical analysis of significant differences between groups. (NS = Not significant; + = p > 0.05; ++ = p > 0.01).

**Figure 6:**
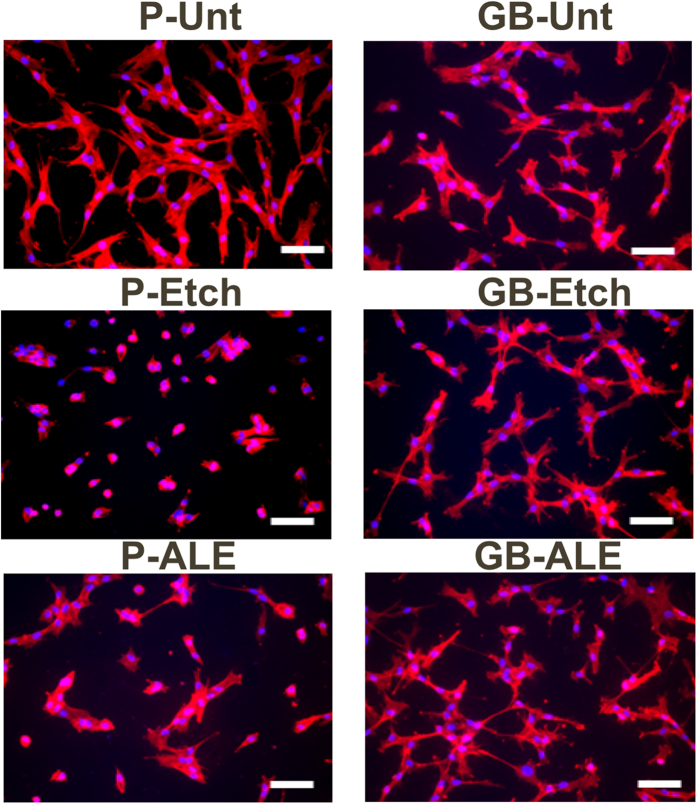
Fluorescent merged staining images of Phalloidin-TRITC stained actin filament, DAPI stained nuclei of hMSCs cultured on treated substrates. (Scale bar = 100 μm).

In order to determine the bone promoting capacity of the alendronate SAM coated Ti6Al4V substrates, hMSC were cultured on the P-Unt substrate (control) and variously treated samples. The effect of surface wettability, roughness and nano-topography in promoting osseointegration of titanium implants has been studied extensively^26^,^27^,^28^, in particular, it has been shown that a roughness of between 1 and 5 μm induces a positive effect on protein adsorption, collagen synthesis and maturation of osteoblasts^29^. Despite GB-Unt at 3 h, there is a highly significant decrease in hMSC attachment for all of the treated substrates when compared to the P-Unt control, as shown in Fig. 5. It is suggested that this is due to the increased SFE of the modified Ti6Al4V that then reduces the adhesion of binding proteins and other adhesive mediating ECM components thereby reducing the attendant cell response. However, despite the reduction in attachment at the early incubation period, cells were able to proliferate on the treated surfaces after more prolonged time periods. Results for the corresponding cell viability assay are provided in Fig. 5, which indicate that the cell population values on the treated Ti6Al4V surfaces are comparable to those observed for P-Unt Ti6Al4V surfaces and the maximum confluency is reached at day 7 for all substrates showing no significant differences in cell viability form day 7 to 14. However, the cells cultured on P-ALE and GB-ALE show a reduction in viability across the total culture period. Figure 6 shows the fluorescence images obtained for stained cell populations on the samples which confirm the amount of attachment for the SAM coatings. These data also show differences in cell size and shape for the cells cultured on both the SAM coating and the alkali-etched substrate surfaces, which is attributable to the change on surface chemistry due to the presence of NH_2_ and OH hydrophilic moieties form alendronate.

Alendronate has been shown to induce osteogenesis in MSCs as well as having the ability to promote bone formation in osteoprogenitor and osteoblast cells both *in vitro* and *in vivo*^30^. Therefore, it is our assumption that the reduction in rate of proliferation seen here, particularly for early time points (3 and 24 hours), is attributable to the changes in cell division that occur due to the initiation of differentiation. However, previous studies have demonstrated that either there is no change in rate of proliferation following treatment with alendronate on tissue culture plastic or that a slight increase occurs^31^. This suggests that alendronate alone is not the cause of the reduction in proliferation and viability observed of the cells cultured on the P-ALE and GB-ALE surfaces in this work whereas previous reported studies on titanium modified surfaces demonstrated that synergic effects of roughness and surface free energy can decrease the cell attachment capability and induce round shaping and size reducing of cells^29^,^32^,^33^.

**Figure 7:**
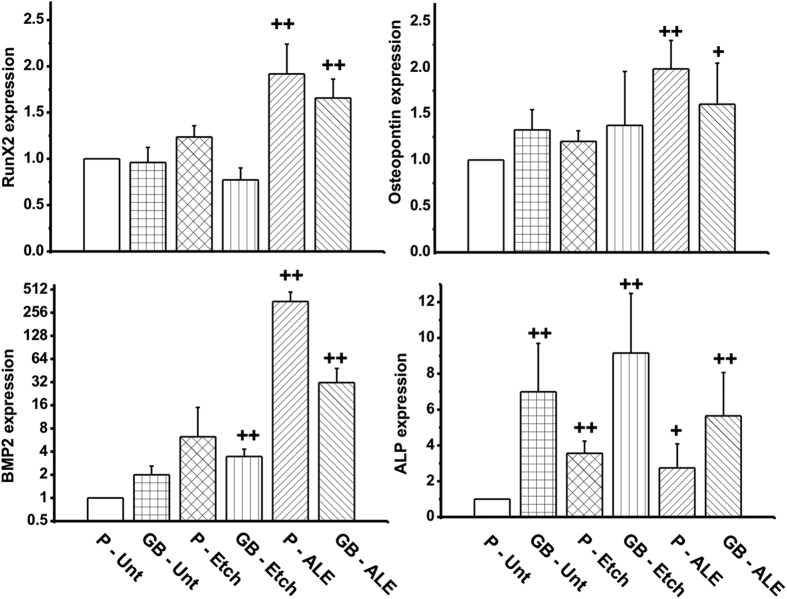
Quantitative qPCR determination of skeletal early markers expression for hMSC cultured on treated substrates for 14 days. Values have been normalized and statistical analysis of significant differences between groups with respect to *P-Unt* (NS = Not significant; + = p > 0.05; ++ = p > 0.01).

In this study, alendronate SAM surface treatment positively contributed to osteogenesis of hMSC as evidenced by upregulation in the expression of early markers of osteogenic differentiation at the time point of middle stage of stimulated osteogenic differentiation of hMSC. These results are illustrated in Fig. 7 and show a significant increase in the expression of Runx2, osteopontin (OPN), alkaline phosphatase (ALP) and BMP2 activity at day 14, for hMSCs cultured in presence of the alendronate monolayers when compared to P-Unt substrate indicating their potential to directly promote osteogenic activity.

It can be observed the activation level of Runx2 and OPN show a similar trend showing only a significant increases when alendronate is present. Nevertheless, the level of ALP expression is also affected by the topography of the substrates as evidenced by the higher expression on GB-Unt and GB-Etch samples. This observation is in accordance with previous studies where it has been shown that moderate roughness (>1 μm) and the presence of residual bioglass particles form grit-blasting treatments enhance the osteogenic activity and ALP levels^29^,^34^,^35^. Most notably, a dramatic increase in the expression of BMP2 in cells cultured on both P-ALE and GB-ALE samples resulted in more than 300 and 30 fold increases, respectively. BMP2 is vital for osteoblast differentiation, mineralisation *in vitro* and bone formation *in vivo*^36^,^37^. In addition, BMP2 has been used for treatment of bone defects by local delivery or when incorporated within a biomaterial scaffold. However, BMP2 has a short half-life and rapidly cleared from the local site of delivery, hampering the use in clinical application^38^. Thus, the results obtained suggest that the presence of alendronate and related SAMs overcome these drawbacks and therefore the clinical need of local delivery of BMP2 can be reduced or even replaced.

The high level of BMP2 activation in hMSCs after 7 days in contact with the alendronate SAMs treated samples, suggest that not only is there an autocrine action on the cells seeded on these surfaces but that possible paracrine effects are also occurring. This suggests a method for treatment of bone defects via stimulation of host osteoprogenitors or via delivery of mesenchymal cells at the site of implantation based on the inclusion of alendronate SAM coatings.

In conclusion the formation of self-assembling monolayers of alendronate onto Ti6Al4V constitutes a simple approach to anchor bone promoting bisphosphonates from aqueous solution onto titanium medical implants with enhanced osseointegration capacity, thus with high potential to expand this unreported methodology to the next generation of metal implants for orthodontic and dental applications.

## Methods

### Materials

Ti6Al4V alloy sheets were obtained from RTI International Metal Ltd, UK. Bioglass Sylc^®^ (50 μm diameter) was purchased from Denfotex Research Ltd, UK and sodium alendronate was purchased from Sigma Aldrich UK and used without further purification. Isopropanol was of extra pure grade form Acros Organics UK. All other reagents were analytical grade and purchased from Sigma-Aldrich UK.

### Ti6Al4V alloy sample preparation

The various surface treatments and resulting sample group codes used in this study are shown in Fig. 1. Titanium alloy sheets were initially polished using 100 grit abrasive paper and then punched into 10 mm diameter disc samples after which they were polished with abrasive walnut shells (Otec Prazisionfinish GmbH) using a dry electro-polishing machine (EcoMaxi Otec, Prazisionfinish GmbH, Germany). This was followed by a cleaning protocol, which involved soaking in boiling deionized water (DI, >18 MΩ·cm^−1^), rinsing in DI water followed by immersion in isopropanol before drying in air. One group of samples was subjected to grit–blasting with Bioglass^®^ particles using a Velopex machine operating at 0.5 MPa. Samples were located at 1 cm from the blast nozzle and processed until saturation of total sample area was complete. All samples were then ultrasonically cleaned in washing up liquid, DI water and isopropanol. Both the untreated P-Unt and grit-blasted GB-Unt samples were further alkali etched by soaking in 5 M NaOH aqueous solution at 60 °C for 24 h and subsequently rinsed with DI water and isopropanol. The four groups P-Unt, P-Etch, GB-Unt and GB-Etch (Fig. 1, groups T1, T2, T3, and T4 respectively) were stored in an oven at 120 °C prior to use.

SAMs formation was carried out by deposition of 100 μl of an alendronate aqueous solution (10 mM, pH 4) onto P-Etch and GB-Etch titanium discs, placed on a hotplate at 80 °C and left to allow the solvent to evaporate. Subsequently the discs were rinsed in 10^−4^ M HCl aqueous solution and the procedure was repeated four times followed by gently washing with DI water and isopropanol. The two samples groups with alendronate SAMs (P-ALE and GB-ALE) were again stored in an oven at 120 °C.

### Characterization of Titanium alloy substrates

The average Ra of the various Ti6Al4V substrates were analysed using a white light interferometer (Zygi, Middlefields, US) equipped with a 10 Mirau lens. Deposition of alendronate was analysed gravimetrically before and after SAMs preparation and the mean value of weight gain was compared with the calculated after immersion of the substrates in PBS for 14 days. The value reported corresponds to the average of measurements performed on 8 different samples. Surface contact angle was measured using the sessile drop technique and employing liquids (5 μl) with known surface tension: water (γ_l_ = 72.8 mN/m) and methylene iodide (γ_l_ = 51.8 mN/m). The SFE of the samples was calculated by Fowkes’ and Ownes method as per the following equations^39^


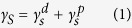



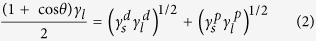


where *θ* is the contact angle, γ_*S*_ and γ_*l*_ are the surface free energy of solid and liquid, respectively, and 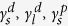 and 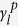 are the dispersive and polar components of surface free energy of solid and liquid, respectively. The values reported correspond to the average of 5 measurements performed on each sample.

The microstructure of the titanium substrates was observed using SEM (Hitachi S3500N) operating at an accelerating voltage of 20 kV. An EDAX analyser (Oxford instrument Micro-analyser) was used to obtain the atomic chemical composition from associated X-ray spectra of the various surfaces.

Raman spectra were recorded for alendronate (control) and P-Unt, P-Etch and P-ALE titanium surfaces using a Renishaw inVia Raman microscope system (Renishaw, Ltd UK) at an excitation wavelength of 785 nm. The system was calibrated using the 520 cm^−1^ Raman peak of a polycrystalline silicon standard and all spectra were collected at room temperature in the range 100 to 3000 cm^−1^ at spatial resolution of 1 μm. Spectra were further analysed using Wire 3.0 software (Renishaw, Ltd UK).

XPS measurements were acquired using a Kratos Axis Ultra DLD spectrometer. Spectra were recorded using monochromated Al Kα X-rays (hυ = 1486.6 eV) generated with the anode operating at 5 kV and 15 mA (75 W). The analysis chamber operating pressure was typically 6.66 × 10^−7^ Pa. Spectra were recorded from three different spots (centre, edge and randomly positioned) with the area of analysis approximately 300 μm × 700 μm and the take-off angle of 90° with respect to the sample normal. Wide energy survey scans were obtained at pass energy of 160 eV and high resolution spectra for O1s, C1s, Ti2p, N1s and P2p regions at pass energy of 20 eV. The Kratos immersion lens charge neutraliser system was used for all samples operating with a filament current of 1.95 A and a charge balance between 3.3 and 3.6 V. Sample charging effects on the measured beam energy positions were additionally corrected by setting the lowest beam energy component of the C1s spectral envelope to 285.0 eV. Quantification of the spectra was carried out by subtracting a linear background and using the peak area for the most intense spectral line of each of the detected elemental species to determine the % atomic concentration.

### Cell culture

Primary hMSC were purchased from Lonza (Slough, UK) and cultured in α-Minimal Essential Medium (MEM), penicillin (50 U/ml), streptomycin (50 μg/ml) (all from Sigma–Aldrich, Poole, Dorset, UK), Glutamax (2 mM) (Invitrogen, Paisley, UK) and 10% foetal bovine serum (FBS) (Sigma–Aldrich) and maintained at 37 °C in a humidified 5% CO_2_: 95% air atmosphere.

### Cell attachment and proliferation

Cells (passage 4−5) were seeded onto the variously prepared experimental and control titanium disks at a density of 2.5·10^4^ per sample. To assess cell attachment, samples were removed from culture after 3 hours and rinsed with PBS to remove non-adherent cells before being transferred to new 24 well plates containing 500 μl of fresh media. Subsequently 100 μl of MTS assay solution (Cell-Titer 96 AQueous One Solution Cell Proliferation assay; Promega, Southampton, UK) was added to each well and cells were incubated for 2 h at 37 °C. 100 μl aliquots of the culture solution from each sample were then transferred to a 96 well plate and the absorbance was measured at 595 nm using a plate-reader (Opsys MR, DINEX Technologies, UK). To assess cell viability, the samples were incubated for 24 h after which they were rinsed and transferred to new 24 well plates containing 500 μl of fresh media. Cells were incubated for specific time points and cell viability was determined using MTS assay.

### Cell morphology and viability

Cells were seeded on the titanium disks at a density of 2.5·10^4^ cells/disk and incubated for 24 h. Phalloidin-TRITC (Sigma) was used to stain for actin filaments and 4′,6-diamidino-2-phenylindole (DAPI) for nuclei. Briefly, hMSC cells on the surfaces were fixed for 15 min with 4% formaldehyde, permeabilised for 10 min with 0.1% Triton X-100 and stained for 40 min with a 0.5 μg/ml phalloidin-TRITC conjugate solution in PBS. Samples were washed with PBS and nuclei were stained with DAPI for 5 minutes and images were acquired using a fluorescent microscope.

### qRT-PCR analysis

Cells were seeded onto the titanium disks at a density of 2.5 × 10^4^ cells per disk and incubated for 24 h after which they were rinsed, transferred to new 24 well plates and placed in culture for 14 days. To assess if osteogenic differentiation had occurred, RNA was isolated after the 14 days of incubation and mRNA expression of markers of differentiation i.e. Runx2, ALP, Osc or osteopontin (Opn) determined by quantitative (q)RT-PCR. Briefly, total RNA was extracted using TRI reagent (Life Technologies) in Phase Lock Gel Heavy tubes (5 prime) according to the manufacturer’s instructions. RNA purity and quantity was assessed by Nanodrop (Fisher Scientific) with A260/A280 1.8–2 considered suitable for further analysis. Any possible contaminating DNA was removed and cDNA prepared from 1 μg RNA using QuantiTect Reverse Transcription Kit (Qiagen) according to the manufacturer’s instructions. qRT-PCR was performed on a Mx3000P real time PCR system (Agilent Technologies) using iTaq SYBR Green qPCR Master mix (Bio-Rad) and primer pairs as follows (5′ to 3′); Runx-2 (AATGGTTAATCTCCGCAGGTC and TTCAGATAGAACTTGTACCCTCTGTT); ALP (AACACCACCCAGGGGAAC and TGGCATGGTTCACTCTCGT); BMP-2 (CGCTCTTTCAATGGACGTGT and GCAGCAACGCTAGAAGACAG); osteopontin (GCCGAGGTGATAGTGTGGTT and TGAGGTGATGTCCTCGTCTG). RPL13a was used as an invariant housekeeping gene (GGATGGTGGTTCCTGCTG and TGGTACTTCCAGCCAACCTC). The (q)RT-PCR conditions consisted of 1 cycle of 95 °C for 3 min and 40 cycles of 95 °C for 10 sec and 60 °C for 20 sec.

### Statistical analysis

All the experiments were performed with four samples per group and mean values and standard deviations are reported. A one-way (ANOVA) and Tukey’s post hoc test were used to determine statistically significant differences (p < 0.05 (+) and p < 0.001 (++)) between the means of different groups.

## Additional Information

**How to cite this article**: Rojo, L. *et al*. Self-assembled monolayers of alendronate on Ti6Al4V alloy surfaces enhance osteogenesis in mesenchymal stem cells. *Sci. Rep*. **6**, 30548; doi: 10.1038/srep30548 (2016).

## Supplementary Material

Supplementary Information

## Figures and Tables

**Table 1:** Surface properties of Ti6Al4V substrates.

Sample	Roughness (μm)	Contact angle		SFE (nM/m)	γD (mN/m)	γP (mN/m)
		H_2_O	CH_2_I_2_			
P-Unt	0.16 [0.05]	41.4 [2.78]	23.8 [5.11]	67.0	47.5	19.5
P-Etch	0.26 [0.04]	27.2 [3.69]	22.1 [3.62]	74.1	48.1	26.0
P-ALE	0.25 [0.04]	13.2 [6.16]	15.6 [6.58]	79.5	49.9	29.6
GB-Unt	1.01 [0.13]	41.8 [0.5]	38.1 [4.3]	63.4	41.4	22.0
GB-Etch	1.07 [0.07]	22.8 [2.5]	20.5 [0.3]	76.0	48.6	27.4
GB-ALE	1.09 [0.05]	16.0 [4.7]	19.7 [2.4]	72.3	48.8	29.5

Mean values (n = 4). Standard deviation in square banquets. SFE = Surface free energy; γD and γD = SFE dispersive and polar components respectively.

**Table 2:** % concentration of nitrogen (N-1s) and phosphorous (P-2p) on the surfaces of the Ti6Al4V alloy substrates as determined by XPS.

Substrate	N-1s	P-2p	N/P
P-ALE	2.33	4.83	0.53
GB-ALE	2.51	4.92	0.51
Alendronate*	3.7	7.4	0.50

^*^Based on molecular formula (C_4_H_13_NO_7_P_2_).
