# Supplementary material for: Self-assembled monolayers of alendronate on Ti6Al4V alloy surfaces enhance osteogenesis in mesenchymal stem cells
Source: Sci Rep. 2016 Jul 29;6:30548. doi: 10.1038/srep30548 (PMC4965747; doi:10.1038/srep30548)
Supplement: Supplementary Information [file srep30548-s1.pdf]

Self-assembled monolayers of alendronate on Ti6Al4V alloy surfaces enhance osteogenesis in mesenchymal stem cells

Luis Rojo Borzo Gharibi, Robert McLister, Brian J Meenan and Sanjukta Deb

Supplementary info

a

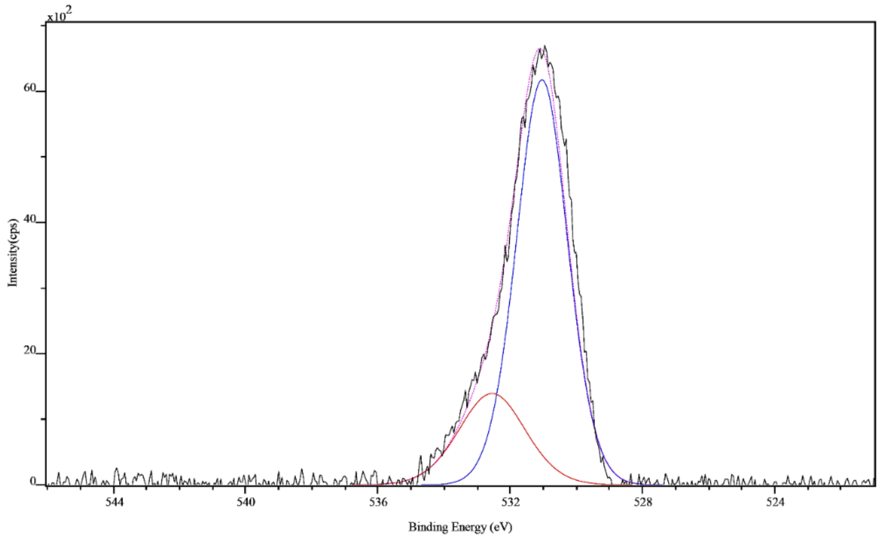

b

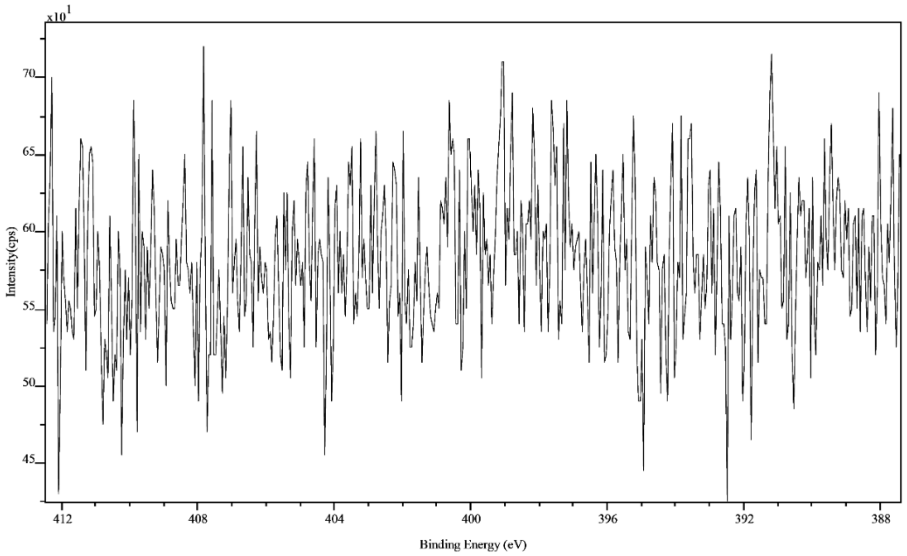

c

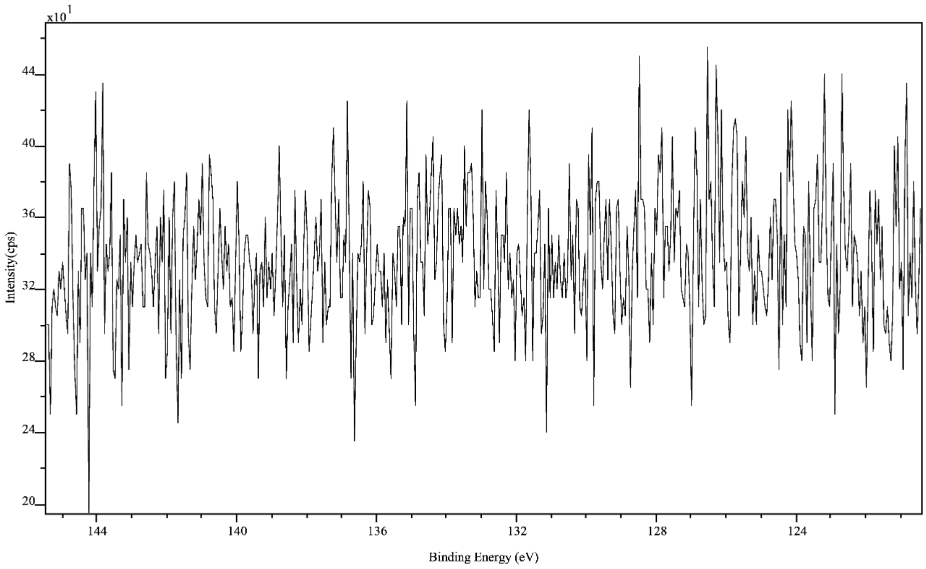

Figure S1. High-resolution XPS spectra of a: O 1s, b: N 1s, c: P 2p of P-Etch samples.

a

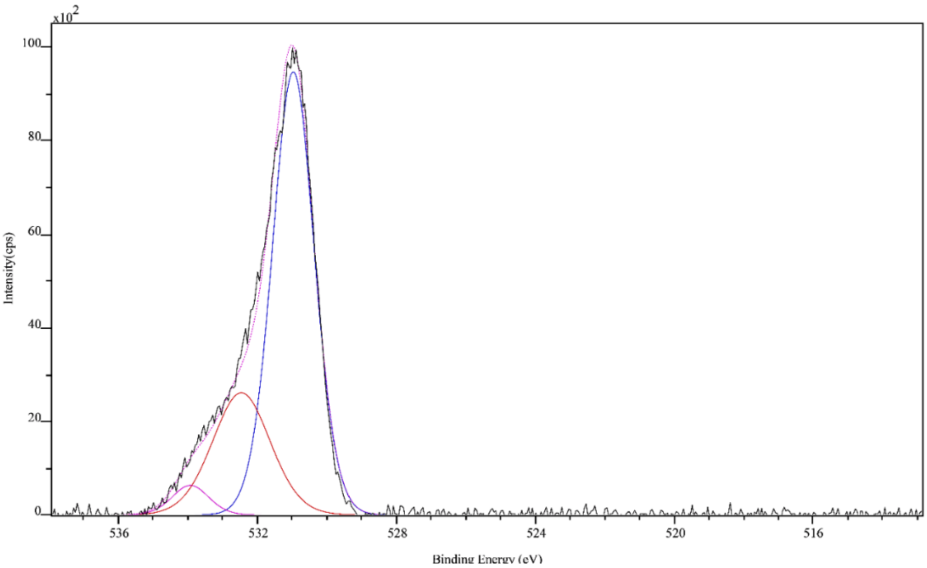

b

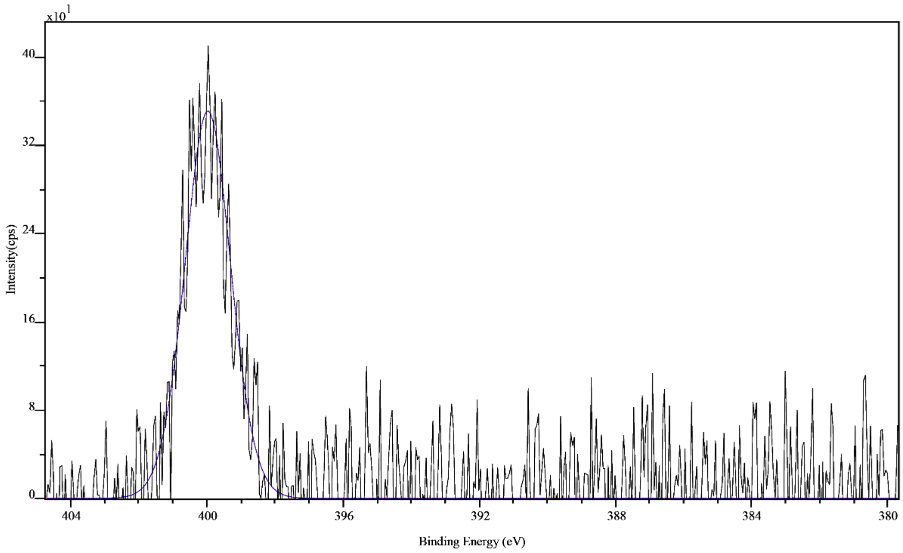

c

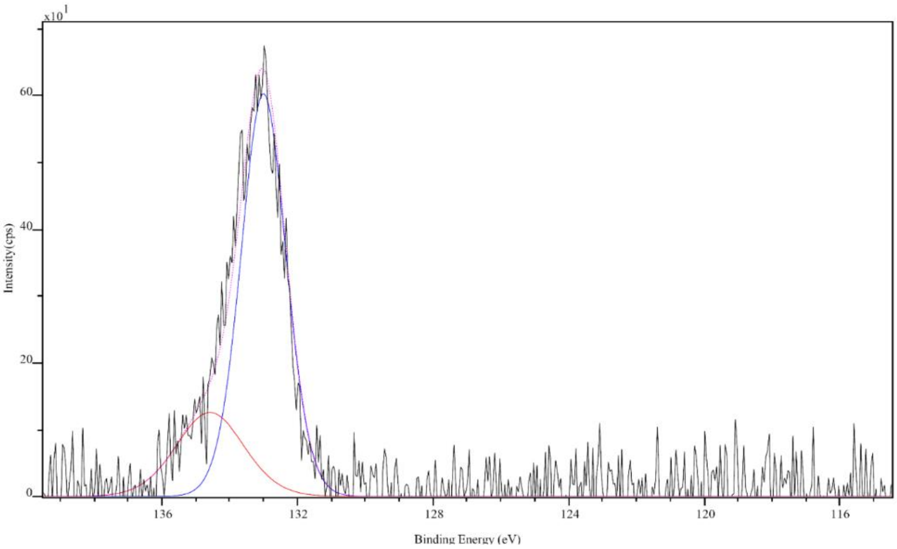

Figure S2. High-resolution XPS spectra of a: O 1s, b: N 1s, c: P 2p of P-ALE samples.
